# Advances in Hydrogel-Based Drug Delivery Systems

**DOI:** 10.3390/gels10040262

**Published:** 2024-04-13

**Authors:** Boya Liu, Kuo Chen

**Affiliations:** 1 Division of Hematology/Oncology, Boston Children’s Hospital, Boston, MA 02115, USA; 2 Department of Pediatrics, Harvard Medical School, Boston, MA 02115, USA; 3 Department of Polymer Science and Engineering, University of Massachusetts, Amherst, MA 01003, USA

**Keywords:** hydrogel, drug delivery, oral, injectable, topical, ocular

## Abstract

Hydrogels, with their distinctive three-dimensional networks of hydrophilic polymers, drive innovations across various biomedical applications. The ability of hydrogels to absorb and retain significant volumes of water, coupled with their structural integrity and responsiveness to environmental stimuli, renders them ideal for drug delivery, tissue engineering, and wound healing. This review delves into the classification of hydrogels based on cross-linking methods, providing insights into their synthesis, properties, and applications. We further discuss the recent advancements in hydrogel-based drug delivery systems, including oral, injectable, topical, and ocular approaches, highlighting their significance in enhancing therapeutic outcomes. Additionally, we address the challenges faced in the clinical translation of hydrogels and propose future directions for leveraging their potential in personalized medicine and regenerative healthcare solutions.

## 1. Introduction

**Figure 1:**
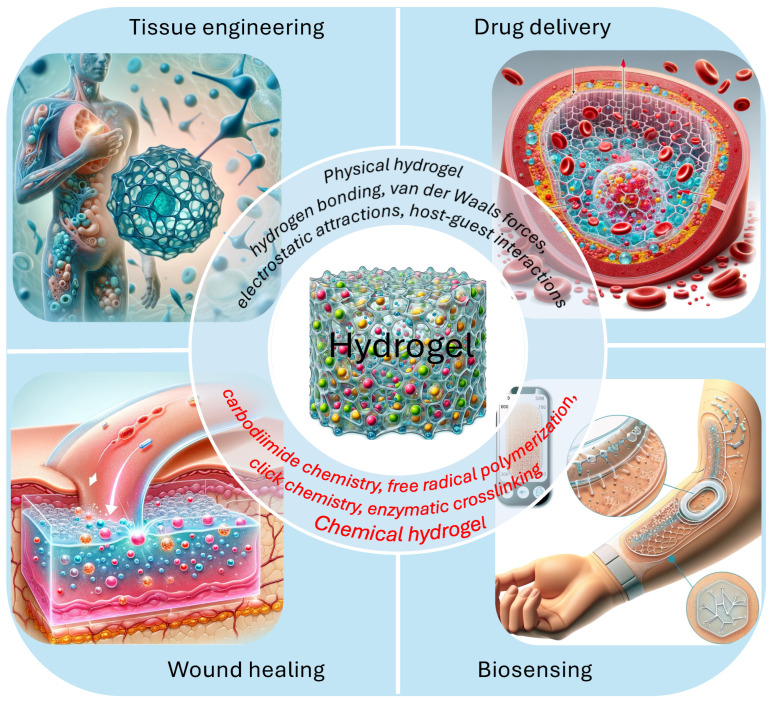
Illustration of hydrogel classification based on cross-linking methods and their biomedical applications.

Hydrogels, characterized by their unique three-dimensional networks of hydrophilic polymers, have emerged as a cornerstone in the advancement of biomaterial science, revolutionizing applications across a broad spectrum of biomedical fields [1,2,3,4,5,6,7,8]. These networks, capable of absorbing and retaining substantial volumes of water, are distinguished by their remarkable ability to swell without dissolution, maintaining structural integrity through chemical or physical cross-linking mechanisms [9,10]. This intrinsic property allows hydrogels to mimic the physicochemical aspects of the natural extracellular matrix, making them particularly suited for applications in drug delivery systems [7,9,11,12,13,14,15,16,17,18,19], tissue engineering [20,21,22,23,24], wound healing [25,26,27,28], and beyond, as illustrated in Figure 1. The initiation of hydrogel research and its expansion into biomedical sciences exemplify a trajectory of innovation, highlighting the versatility of these materials in solving complex biological challenges and their role in advancing biomedical solutions.

The foundation of hydrogel technology was laid through the pioneering work of Wichterle and Lim, who produced poly(hydroxyethylmethacrylate) (pHEMA) hydrogel in the 1960s [29], marking the inception of hydrogels as biomaterials. Since then, the field has witnessed exponential growth, driven by advancements in polymer science and a deepening understanding of biological interfaces [1,30]. In addition to the comprehensive experimental investigation of hydrogels in biomedical applications, particularly in drug delivery, theoretical frameworks and computational modeling have been instrumental in shedding light on the structural dynamics of hydrogels [31,32,33,34,35,36,37]. They provide valuable insights into characteristics such as elasticity, porosity, and mesh size. These predictive models, based on thermodynamic principles, have enabled the design of hydrogels with customized properties for targeted biomedical uses. Hydrogels can be broadly categorized into natural, synthetic, and semisynthetic, based on their origin and the nature of their cross-linking mechanisms [38,39,40]. This classification highlights the need to balance mechanical strength with biodegradation rates for optimal biocompatibility and functionality. To be biocompatible, hydrogels must be non-toxic and not trigger adverse immune reactions. Functionality requires adjusting physical properties like porosity, swelling, and strength to suit specific applications, from drug delivery to tissue engineering. The evolution of hydrogel polymerization techniques, including the development of homopolymers, copolymers, and interpenetrating networks, further demonstrates the versatility of these materials, enabling the customization of hydrogel properties to meet the demands of specific biomedical applications.

The ability of hydrogels to respond to external stimuli, such as chemical, physical, or biological factors, has unveiled novel opportunities for the creation of intelligent materials [9,14,39,41]. This characteristic is pivotal in the conception of dynamic hydrogel systems that can adjust to varying physiological conditions, paving the way for advanced applications in smart drug delivery systems, adaptive tissue scaffolds, and responsive biomedical devices. The introduction of injectable hydrogels, characterized by their shear thinning and self-healing properties, represents significant progress towards the development of less invasive therapeutic modalities [42,43]. These hydrogels present a promising alternative to conventional surgical techniques, offering targeted drug delivery and tissue repair directly at the site of action. Nonetheless, the path towards the clinical translation of these materials is beset with challenges, particularly the imperative to mitigate adverse immunogenic responses and ensure the removal of deleterious by-products from the cross-linking process [44]. Recent advancements in nanotechnology have precipitated the emergence of nanogels, a novel class of hydrogel-based nanomaterials with promising prospects in drug delivery and tissue engineering [45,46]. Alongside progress in 3D bioprinting [47,48], hydrogels are now at the vanguard of fabricating tissue constructs with unprecedented precision and complexity, heralding a new epoch in regenerative medicine and tissue engineering.

In the realm of drug delivery systems, hydrogels encounter limitations pertaining to biocompatibility, safe assembly, and controlled drug release [49]. These materials must be constructed from biocompatible constituents to circumvent immune reactions and assembled utilizing non-toxic chemistries in aqueous conditions at physiological pH. Despite the availability of suitable synthetic and natural polymers, the assurance of controlled therapeutic release, whether through physical constraints or drug-material affinity, remains a formidable challenge. Advances in polymer science have led to the creation of biopolymers that offer a biodegradable and biocompatible framework for hydrogels, augmenting their utility in targeted and efficient drug delivery. However, achieving the requisite mechanical stability, biodegradability, and target specificity while ensuring safety and efficacy in the physiological milieu highlights the ongoing challenges in the design of hydrogel-based drug delivery systems.

In this review, we begin by categorizing hydrogels based on their cross-linking methods, then proceed to explore the critical properties of hydrogels that influence drug delivery, integrating both theoretical insights and experimental results. This encompasses assessments of mesh size, swelling behavior, porosity, microstructures, mechanical properties, and the degradation features of biodegradable hydrogels. Subsequently, we address recent advancements in hydrogel applications, particularly in drug delivery, underscoring their significance in enhancing therapeutic outcomes. Furthermore, we delve into the current challenges and future directions in the utilization of hydrogels for drug delivery. In summary, this review underscores the transformative potential of hydrogels in biomedical engineering, spotlighting their evolving applications in drug delivery and the critical need for ongoing innovation to address existing challenges and unlock new avenues for therapeutic intervention.

## 2. Classification of Hydrogels

**Figure 2:**
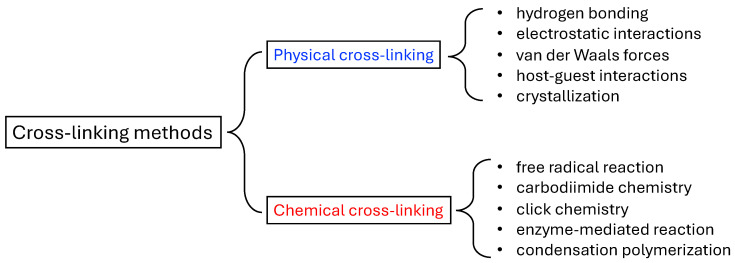
Classification of hydrogels based on the cross-linking formation mechanisms.

The classification of hydrogels can be based on various criteria, including their source, cross-linking methods, composition, degradability, stimuli responsiveness, and ionic charge. In this context, we categorize them into physical and chemical hydrogels, distinguished by their cross-linking formation mechanisms, as illustrated in Figure 2. Hybrid gels featuring both physical and chemical cross-linking are not individually classified here. We proceed to select and review various representative hydrogels characterized by diverse gelation mechanisms.

### 2.1. Physcial Hydrogel

Physical hydrogels are synthesized from low-molecular-weight compounds or polymers through non-covalent interactions such as hydrogen bonding [50,51,52,53], van der Waals forces [11], electrostatic attractions [54,55,56], and specific host-guest interactions [57,58,59]. These reversible interactions allow physical gels to undergo gel-to-sol transitions in response to environmental stimuli like temperature, pH, and ionic strength, endowing them with adaptability and responsiveness. In contrast to chemical gels, which rely on permanent covalent bonds, physical gels form transient networks through weak, albeit numerous, physicochemical bonds, granting them properties such as softness, reversibility, and sensitivity to external stimuli. These features make physical gels particularly suitable for applications requiring sensitivity to environmental changes, such as in drug delivery and tissue engineering scenarios. Numerous physical hydrogels have been designed for use in drug delivery applications, with several notable examples listed below.

Yoshimura et al. introduced biodegradable hydrogels synthesized by reacting starch with succinic anhydride (SA), using 4-dimethylaminopyridine as an esterification catalyst in DMSO or water, followed by NaOH neutralization [51]. The formation of the starch-SA hydrogel is attributed primarily to the regeneration of hydrogen bonds during the dialysis process. These hydrogels, with substitution degrees ranging from 0.1 to 1.4, demonstrate maximum water absorbencies of up to 120 g-water/g-dry gel. Notably, hydrogels produced in DMSO showed superior substitution degrees and absorbencies due to the reduction of SA hydrolysis, suggesting their suitability for biomedical and agricultural uses given their biodegradability and efficient water absorption.

Lu et al. crafted an injectable hydrogel through the physical blending of carboxymethyl hexanoyl chitosan and hyaluronic acid, exploiting supermolecular interactions to form a network capable of sustained therapeutic release [58]. This injectable modality, paired with its controlled release capabilities, is poised for localized drug delivery and regenerative medicine applications. Tran et al. devised a syringeable hydrogel from β-cyclodextrin and mixed micelles for methotrexate delivery [59], utilizing host-guest interactions for hydrogel formation, which enhances drug delivery efficiency, showcasing the innovative use of host-guest chemistry in developing functional biomedical materials.

Sun et al. explored a novel hydrogel formulation integrating nanostructured lipid carriers (NLC) with chitosan-tripolyphosphate (chitosan-TPP) hydrogel beads, leveraging hydrophobic interactions for efficient encapsulation of hydrophobic active substances [60]. The chitosan-TPP hydrogel forms through electrostatic interactions between the phosphate groups of TPP and the protonated amino groups of chitosan. This strategy not only improves bioavailability and controlled drug release but also holds significant promise for enhancing topical drug delivery systems, illustrating the potential of hydrophobic interactions in innovative hydrogel designs.

### 2.2. Chemical Hydrogel

Chemical hydrogels, synthesized through covalent bonding of polymers, leverage chemically active motifs for crosslinking, utilizing a variety of methods such as carbodiimide chemistry [61,62,63], free radical polymerization [29,64,65,66,67,68,69,70,71,72,73], and click chemistry [74,75,76,77,78,79,80]. These approaches offer chemical hydrogels enhanced matrix stabilization and greater control over gel formation, enabling a higher degree of flexibility and spatiotemporal precision than physical gels. Specifically, enzymatic crosslinking, utilizing biocompatible enzymes like peroxidases and transglutaminases, presents a favorable method for creating biocompatible, nonimmunogenic hydrogels suitable for tissue engineering, drug delivery, and regenerative medicine (TERM) applications due to its mild reaction conditions and minimal cytotoxicity [81,82]. Additionally, electron irradiation techniques have emerged, providing efficient and precise crosslinking capabilities that expand the possibilities for hydrogel customization [83,84].

The pioneering synthesis of pHEMA hydrogels by Wichterle and Lim [29], achieved through free radical polymerization, highlights the potential for integrating acrylate derivatives into biopolymers such as dextran [64], albumin [69,70], starch [68,71], and hyaluronic acid [72,73], transforming them into cross-linked hydrogels. Similarly, high-energy radiation, including gamma rays and electron beams, facilitates the transformation of vinyl polymer solutions into hydrogels or initiates the polymerization of monofunctional acrylates with cross-linkers, forming hydrogels [65,66,67]. This process involves radical generation from carbon-hydrogen bond breaking or water molecule radicals, which then recombine to create covalent cross-links. Specifically, polyvinyl alcohol, polyethylene glycol, and polyacrylic acid are capable of undergoing such radical reactions, facilitating the formation of hydrogels.

Furthermore, the Click chemistry reaction, catalyzed by monovalent copper to form triazole rings from azides and alkynes, stands out for its efficiency and by-product-free nature, making it ideal for in situ hydrogel formation [74,76,77,78]. This reaction is particularly suited for modifying polymers like polyethylene glycol, hyaluronic acid, gelatin, and peptides with azido or alkynyl groups for cross-linking, facilitating the creation of enzyme-degradable and cell-adhesive hydrogels. To avoid the toxicity associated with copper ions, copper-free Click reactions utilizing cyclooctyne derivatives have been developed, offering safer cross-linking alternatives [75,79,80].

Sperinde et al. used enzymes for hydrogel synthesis, developing a system based on tetrahydroxyl polyethylene glycol (PEG) functionalized with glutamine [85]. By incorporating glutaminase into a solution of glutamine-functionalized tetrahydroxyl PEG and poly(lysine phenylalanine), they catalyzed the formation of amide bonds between the glutamine and lysine groups, effectively cross-linking the polymers. This enzymatic approach, particularly with the calcium ion dependence of transglutaminase, allows for the design of stimulus-responsive gelation systems, featuring the depth of chemical versatility in hydrogel synthesis for targeted applications.

## 3. Characterization of Hydrogels

This section delves into the crucial properties of hydrogels, such as mesh size, swelling behavior, porosity, microstructure, mechanical strength, and degradability. We start by examining why these characteristics are vital for the effectiveness of hydrogel-based drug delivery systems. Next, we investigate the common techniques used to measure these physical properties. We then highlight how the manipulation of these properties can enhance hydrogel performance. The pros and cons of these characterization techniques are neatly summarized in Table 1. Recently, Denzer et al. have offered an extensive overview of the methods employed in hydrogel characterization [86].

### 3.1. Mesh Size and Swelling Behavior

The swelling behavior of hydrogels in drug delivery significantly impacts drug release and diffusion, making it a crucial factor in their application. Hydrogels, with their crosslinked polymer networks, facilitate the movement of liquids and solutes through open spaces or meshes. The mesh size (ξ) of hydrogel used in drug delivery, typically less than 200 nm, is pivotal in determining the interaction between the drug and the polymer network, thereby controlling the diffusion of drugs [12]. However, it is important to note that hydrogels often display a wide range of mesh sizes due to network heterogeneity and polymer polydispersity. This variability is especially pronounced in hydrogels formed through free-radical polymerization. Achieving a uniform mesh size is possible by using symmetrical tetrahedron-like macromeres of identical size for gelation, leading to hydrogels with a more uniform mesh size [87,88,89]. For smaller drug molecules, release is primarily governed by diffusion, allowing for their free movement within the network. This process is influenced by factors such as the drug size and the viscosity of a medium, as dictated by the Stokes–Einstein equation. Typically, drugs are composed of small molecules, resulting in their entropic effects being relatively minor when trapped within the gel network, especially in comparison to the more complex dynamics of the embedded polymers [90,91,92]. When mesh and drug sizes are similar, steric hindrance slows diffusion, enabling prolonged release. A small ratio between mesh size and drug leads to entrapment until the network degrades or expands.

Characterizing the swelling ratio (Q) of hydrogels can be approached through various methodologies, each with its own set of advantages and challenges. A prevalent and straightforward method is to dry or lyophilize the hydrogel sample with a dry weight, $W_{d}$, and immerse it in a substantial volume of water or specific buffer solutions for predetermined durations such as 16, 24, or 48 h [10,25,26,51,62,64,65,68,72,78,93,94], followed by filtration and measurement of the swollen hydrogel’s weight ($W_{s}$) to determine the degree of swelling using the formula:

(1) $$Q=\frac{W_{s}-W_{d}}{W_{d}}$$

The immersion time is critical and varies based on the time to reach swelling equilibrium, which presents a limitation due to the difficulty in precisely determining this point. Furthermore, the filtration step introduces variability based on the mesh used and the operator’s technique, potentially complicating measurements for very soft hydrogels that might obstruct the mesh. An alternative method, particularly suitable for hydrogels with isotropic network structures [54,85,95], calculates the volumetric swelling ratio from the thickness swelling ratio as $Q={\left(t/t_{0}\right)}^{3}$, where *t* is the thickness of the swollen gel and $t_{0}$ is its original thickness. This method directly correlates isotropic swelling behavior with volumetric changes, emphasizing the importance of precise measurement techniques for thickness determination.

The mesh size (ξ) of the hydrogel can be characterized through two distinct methods [65,96,97,98,99,100]. The first method assesses the cross-link density, or the number of monomers positioned between cross-linkers. This measurement can be derived from mechanical or rheological testing and incorporates the swelling ratio into the ξ estimation. This integration leverages principles from equilibrium-swelling theory and rubber-elasticity theory, particularly relevant for highly swollen hydrogels:

(2) $$\xi =Q^{1/3}{\left(2C_{n}N\right)}^{1/2}l$$

where $C_{n}$ is the Flory characteristic ratio, *N* is the number of monomers between two adjunct cross-linkers, and *l* is C-C bond length. The effective cross-linking density could be determined by the elasticity or compressive modulus from mechanical or rheological testing, which were well described in entensive studies [101]. Rheological analysis, notable for its independence from sample size or shape, emerges as more precise and reproducible than mechanical testing. The second method involves determining the correlation length through various scattering techniques, such as static light scattering, small-angle X-ray scattering, and small-angle neutron scattering, which combines the Lorentzian and power law model fitting to the scattering curves and provides the ξ network calculated as [88,89,90,91,98,99]:

(3) $$I\left(q\right)=\frac{I(0)}{1+q^{2}\xi^{2}}+\frac{A}{q^{n}}$$

where *q* is the scattering vector, *I*(*0*) is the intensity at *q* = 0, A is a constant, and *n* is the power law exponent in the low-*q* region. While incident light in scattering techniques, encompassing laser, X-ray, and neutron, spans a broad range of scattering vectors or detection lengthscales from 1 to 1000 nm, the utilization of these methods for determining mesh size necessitates sophisticated instrumentation and relies heavily on fitting models.

Controlled swelling of hydrogels, where the mesh size expands, offers another avenue for drug release. Swelling is influenced by a balance between network deformation forces and osmotic water absorption, responsive to external conditions like temperature, pH, and ionic strength. Lots of hydrogels have been engineered to leverage these environmental changes for effective drug delivery. Recently, Han et al. have introduced a dual pH-responsive hydrogel actuator tailored for the delivery of lipophilic drugs, drawing inspiration from the movement of Drosera leaves [102]. This system encapsulates drugs within a capsule comprising two pH-responsive hydrogel layers that release drugs via a “turn on” motion in specific pH settings, thereby increasing both the scope of lipophilic drugs and their loading efficiency. Similarly, Wang et al. have developed a biomimetic system that triggers insulin release in response to glucose, employing a hydrogel infused with β-cyclodextrin and insulin [103]. This innovative setup specifically reacts to D-glucose, offering precise and on-demand insulin release, and differentiates D-glucose from similar isomers, enhancing blood glucose control for up to 12 h in Type I diabetic mice. However, the effectiveness of swelling-controlled systems is limited by the slow diffusion of water, particularly in larger hydrogels. Strategies to enhance response times include reducing hydrogel size or incorporating quick-swelling layers.

The theoretical analysis of hydrogel swelling, incorporating models from Brannon-Peppas and Peppas [31], leverages the Flory-Huggins theory and rubber elasticity theory, including ionic interactions, to describe pH-sensitive hydrogels’ equilibrium swelling behavior. Recent work by Jia and Muthukumar further elucidates the theory of charged hydrogels [37], emphasizing the significance of electrostatic and hydrodynamic interactions in determining hydrogel properties. These theories and models facilitate the design of hydrogels with tailored swelling behaviors and drug release profiles, essential for optimizing their biomedical applications.

### 3.2. Porosity and Microstructures

Porosity (*P*) and microstructure are foundational to the biomedical utility of hydrogels, as they dictate the internal void spaces and structural arrangement [12,104,105,106]. These properties are crucial for fluid dynamics, including drug delivery and nutrient-waste exchange in tissue engineering contexts, and are influenced by polymerization techniques and cross-linking density. The microstructure, encompassing pore size, shape, and distribution, is shaped by synthesis conditions and affects the mechanical properties, biocompatibility, and biodegradability of hydrogels. Optimizing porosity and microstructure is therefore vital for designing hydrogels that mimic the extracellular matrix, promoting cell growth and migration, enabling precise drug release, and ensuring predictable degradation for seamless tissue integration. The creation of interconnected porosity within polymeric hydrogels represents a significant innovation, improving performance through enhanced solvent transport via convective flow rather than simple diffusion [105]. This feature is especially critical in the biomedical application of hydrogel, where tailored porosity and pore size distribution facilitate drug release. Various fabrication methods, including foaming, phase separation, in situ crosslinking polymerization, particulate leaching, freeze-drying, and reverse casting, have been employed to achieve hydrogels with precise porosity, significantly impacting their porosity and microstructure. Research indicates that the swelling and degradation behaviors of hydrogel are profoundly influenced by its porous characteristics [94,107], such as void fraction and pore interconnectivity. Moreover, the drug delivery efficacy of hydrogel is largely determined by its water content and porosity, which affect solute absorption and diffusion. Advanced production techniques, like solvent casting, electrospinning, fused deposition modeling, and 3D printing, have been investigated for crafting porous scaffolds, underscoring the necessity for innovative approaches in hydrogel development to optimize their porosity and microstructure for biomedical applications [47,48].

The common method used in the calculation of the porosity of the hydrogel is based on the weight of the hydrogel before and after drying [101,108], incorporating the previously mentioned swelling ratio as follows:

(4) $$P=\frac{\left(W_{s}-W_{d}\right)/\rho_{w}}{W_{d}/\rho_{p}+\left(W_{s}-W_{d}\right)/\rho_{w}}=\frac{Q/\rho_{w}}{1/\rho_{p}+Q/\rho_{w}}$$

where $\rho_{p}$ is density of water and $\rho_{w}$ is density of polymer. This approach to assessing porosity is more straightforward than alternative techniques, which include the diffusion of probes within the hydrogel, measuring water content through differential scanning calorimetry (DSC), and testing the permeation of probe solutes. Other complex methods such as mercury porosimetry, gas pycnometry [104], and liquid extrusion porosimetry also fall into this category. The microstructure of hydrogels is typically characterized using a variety of microscopy techniques, including optical microscopy, scanning electron microscopy (SEM) [10,22,25,26,58,59,62,63,78,106,108], transmission electron microscopy (TEM) [22,86], and atomic force microscopy (AFM) [86], which elucidate surface morphology and topographical details. For a more in-depth internal analysis, micro-computed tomography (Micro-CT), or X-ray microtomography, serves as a high-resolution, nondestructive method to evaluate pore size, distribution, and the directional orientation of pores, providing a comprehensive view of the internal structure. The advantages and disadvantages of these methods are detailed in Table 1.

### 3.3. Mechanical Properties

The mechanical properties of hydrogels, such as tensile strength, elasticity, and viscoelasticity, play a pivotal role in their application for drug delivery, enabling them to mimic the mechanical environment of natural tissues, support cellular functions, and withstand dynamic physiological stresses. These properties are essential for the successful integration of hydrogels into biological systems, facilitating sustained and controlled drug release. Mechanical deformation of hydrogels, through methods like mechanical force, ultrasound, and magnetic fields, offers a strategic avenue for drug release [109,110,111]. This approach can alter the network structure, increase mesh size, and induce convective flow within the hydrogel, enabling pulsatile release patterns that can mimic biological signaling processes, such as the release of insulin postprandially.

To accurately assess the mechanical properties of hydrogels, researchers utilize rheological measurements and uniaxial compression tests [10,25,26,50,53,58,59,63,64,66,68,72,78,88,95,100,101,106,110]. Micro-rheology and mechanophores [86,112,113], used to probe the local mechanical properties within the heterogeneous hydrogel environment, also fall into this category. Rheological measurements, involving deformation and frequency sweep tests, determine the viscoelastic properties of the hydrogel. This process includes estimating the linear viscoelastic region and subsequently determining the elastic ($G^{\prime }$) and viscous ($G^{\prime \prime }$) moduli. From these values, the aforementioned effective cross-link density can be calculated. Complementary to this, uniaxial compression tests measure the hydrogel’s response to force, providing data on stress, strain, and the compression modulus (*G*). Compared to uniaxial tensile mechanical tests, rheological tests require much less preparation of samples, especially for extremely soft gels. However, preparing uniformly sized samples for mechanical testing can be very challenging and varies significantly from one researcher to another, leading to less consistent reproducibility in experiments.

However, challenges such as the high water content and network heterogeneity of hydrogels often compromise their mechanical stability, posing limitations to their practical utility. To address these issues, research has been directed towards enhancing the mechanical performance of hydrogels through innovative strategies, including the development of double-network hydrogels and the introduction of chemically or ionically cross-linked hydrogels. Noteworthy is the work by Gong et al. [114], which introduced “sacrificial weak bonds” in double network hydrogels to improve mechanical durability. This approach combines a densely cross-linked polyelectrolyte network with a loosely cross-linked polyacrylamide network. The sacrificial breakdown of the former absorbs energy and prevents crack propagation, significantly boosting the toughness of hydrogels.

Advancements continue with the exploration of reversible physical interactions—such as ionic bonds, crystallization, hydrophobic interactions, and hydrogen bonding—to further enhance the resilience of hydrogel networks. A significant breakthrough by Suo et al. involved creating a dual cross-linked double network hydrogel through a one-pot process [115], incorporating sodium alginate and acrylamide with N,N′-methylenebisacrylamide and calcium ions. This innovation leads to a hydrogel that, after photoinitiated polymerization, showcases remarkable extensibility and toughness, is able to stretch more than 20 times its original length, and achieves a tear energy of nearly 9000 J/m^2^. Its notable stretchability, even in the presence of notches allowing for a 17-fold extension from its original length, exemplifies the potential of integrating physical interactions to significantly bolster the mechanical properties of hydrogels for drug delivery and other biomedical applications.

### 3.4. Degradability

Degradability in hydrogels is a key attribute tailored for biomedical applications, allowing these materials to break down into biocompatible by-products that can be safely metabolized or excreted by the human body [12]. This property ensures that hydrogel-based devices, such as drug delivery systems, tissue engineering scaffolds, or temporary implants, perform their intended therapeutic functions and then degrade at a controlled rate to minimize long-term adverse effects. The degradability rate and mechanism—often achieved through hydrolysis or enzymatic degradation—are influenced by the chemical composition of hydrogel, including the nature of the polymeric backbone and the presence of degradable linkages. For example, hydrogels incorporating poly(lactic-co-glycolic acid) or peptides designed to be cleaved by specific enzymes can provide precise control over degradation timescales, aligning with tissue healing processes or drug release profiles [116,117]. Optimizing degradability for biomedical hydrogels is crucial for their successful integration and function in medical treatments, promoting efficient treatment while avoiding the need for surgical removal.

The standard method for quantitatively characterizing the degradability of hydrogels in vitro involves measuring weight loss during the degradation process [25,26,58,62,63,64,65,75,94,118,119]. This encompasses mechanisms such as enzymatic hydrolysis, photolytic cleavage, and ester hydrolysis, or a combination thereof. To conduct this assessment, a hydrogel sample with an initial dry mass ($m_{\mathrm{in}}$) is incubated in a buffer solution under specified degradation conditions for a defined period. Subsequently, the sample is dried to remove water and degraded monomers or oligomers, and then weighed to ascertain the final mass ($m_{\mathrm{af}}$). The percentage of mass loss is calculated using the formula:

(5) $$\mathrm{Mass}\;\mathrm{loss}\;\%=\left(\frac{m_{\mathrm{in}}-m_{\mathrm{af}}}{m_{\mathrm{in}}}\right)\times 100\%$$

Moreover, analyzing the change in content of specific components within the gel network before and after degradation offers further insight into the degree of degradation. Comparative imaging of hydrogel sizes is also qualitatively utilized to estimate the degradability of hydrogels [120,121].

Different biodegradability strategies in hydrogels have been explored for biomedical applications, with two representative examples illustrating this trend. Shmidov et al. conducted a study on how the topology of crosslinkers affects the enzymatic degradation of hyaluronic acid (HA) hydrogels [122]. By employing dendritic and linear elastin-like peptides (ELPs) as crosslinkers and examining their degradation with trypsin, it was found that hydrogels crosslinked with dendritic ELPs degrade more slowly than those with linear ELPs. This difference is attributed to the steric hindrance and unique structure of dendritic peptides, highlighting the influence of crosslinker topology on hydrogel degradation rates. Such insights pave the way for customizing hydrogel properties for drug delivery applications. On another front, Ashley et al. introduced a hydrogel-based drug delivery system engineered for adjustable drug release and degradation rates, employing β-eliminative linkers [123]. These linkers facilitate drug attachment to PEG hydrogels, granting precise control over drug half-lives from mere hours to beyond a year. The method presents a novel way to prolong the therapeutic action of drugs, particularly peptides and proteins, by circumventing the typical renal elimination challenges of circulating carriers. Their work showcases the innovative use of biodegradable hydrogels for developing subcutaneous implants as drug carriers, aiming for significantly extended half-lives for various therapeutics.

## 4. Hydrogel in Advanced Drug Delivery System

Contemporary research employs a variety of delivery methodologies, such as nanoparticles, liposomes, ethosomes, nanocomposites, and more, for the targeted administration of drugs to efficaciously address diseases [124,125,126]. Parallel to these developments, hydrogels have emerged as a focal point of interest due to their exceptional utility in devices designed for therapeutic agent targeting, bioadhesion, and controlled release [4,7]. The distinctive properties of hydrogels, such as their ability to retain substantial amounts of water, their biocompatibility, and their controlled swelling behavior, have significantly contributed to their prominence in drug delivery systems [2,11]. These hydrogel formulations are engineered to facilitate the gradual elution of drugs, thereby sustaining elevated concentrations of the medication in the target area and adjacent tissues for extended periods. This attribute underscores their potential for the systemic administration of a myriad of therapeutic drugs and bioactive compounds [49,127]. Furthermore, hydrogel-based delivery mechanisms have been innovatively devised for oral, injectable, topical, and ocular applications.

### 4.1. Oral Hydrogel-Based Drug Delivery

Oral drug delivery is instrumental for administering a broad spectrum of therapeutic agents aimed at treating both local and systemic diseases [128]. Nevertheless, the oral route presents challenges for the delivery of certain categories of drugs. Peptide and protein therapeutics, for example, are particularly vulnerable to acidic denaturation and enzymatic degradation and face issues related to stability, solubility, and absorption in the gastrointestinal tract (GIT) [129]. Hydrogels offer a promising solution for site-specific delivery within the GIT, safeguarding therapeutic molecules through their complex milieu and facilitating controlled, site-specific drug release.

**Figure 3:**
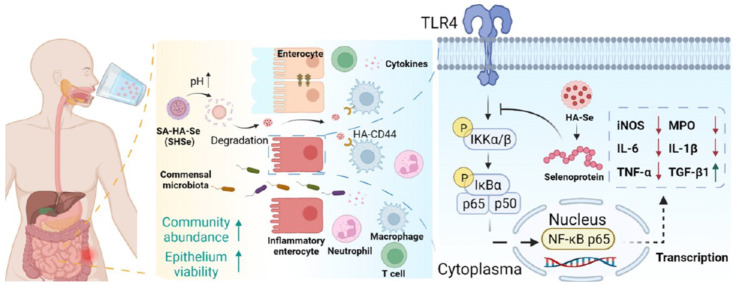
Schematic of SHSe microbead delivery: Upon reaching the intestine, they release HA-Se to target colon inflammation, modulating immune responses and optimizing gut microbiota by altering cytokines, immune cells, and bacterial communities. Adapted with permission from [131]. Copyright *©* 2023, American Chemical Society.

Ji et al. engineered an innovative platform for oral protein delivery by modifying the zeolitic imidazole framework-90 (ZIF-90) with medium-chain lipids (C10) and encapsulating these nanoparticles within sodium alginate [130]. This approach not only efficiently encapsulates proteins, safeguarding them from gastrointestinal degradation, but also enhances mucosal penetration and cellular uptake. Moreover, the modified ZIF-90 nanoparticles are designed to release their payload in response to adenosine triphosphate, enabling targeted delivery to disease-afflicted cells. Ouyang et al. devised a novel strategy for the oral administration of selenoproteins bluethough in situ synthesis of hydrogel microbeads and modulation of cytokine levels and immune cell distribution [131], as shown in Figure 3. These microbeads, created by enveloping hyaluronic acid-modified selenium nanoparticles in a calcium alginate (SA) hydrogel shell, circumvent the obstacles traditionally associated with oral protein delivery. Demonstrated in mouse models of inflammatory bowel disease (IBD), this methodology significantly attenuated proinflammatory cytokine levels, modulated immune cell distributions, and altered the gut microbiota, suggesting its therapeutic potential for diseases linked to intestinal immunity and microbiota.

Hydrogels have also been explored for the precise delivery of antigens, vaccines, DNA, proteins, or peptides [132,133]. Li et al. bluepresent a glucose-responsive nanocarrier system for insulin delivery, encapsulated within a three-dimensional hyaluronic acid hydrogel, demonstrating enhanced oral bioavailability and prolonged hypoglycemic effects in diabetic rats compared to insulin-loaded nanocarriers alone [134]. Zhu et al. reported the development of bluezein/sodium alginate-based core-shell microspheres (Zein/SA/BG) for oral delivery of bioactive glass (BG), which helps prevent premature dissolution of BG in the stomach and significantly reduces intestinal inflammation, promotes epithelial tissue regeneration, and partially restores microbiota homeostasis [135]. Miller et al. presented a self-assembling, pH-responsive nanoparticle systemblue, which were synthesized via nanoprecipitation using pH-responsive copolymers based on poly(methacrylic acid-co-methyl methacrylate)-block-poly(ethylene glycol), offering a promising non-invasive alternative to injections [136]. Touzout et al. proposed a pH-sensitive calcium alginate/polyvinyl alcohol hydrogel bead system for the controlled oral delivery of curcumin, demonstrating its antibacterial and antioxidant capabilities [137]. Despite the inherent challenges of oral drug administration, an array of hydrogel-based systems have been developed to enhance the performance and efficacy of oral drug delivery strategies. Andretto et al. developed hybrid lipid-polymer nanocomposites by integrating bioadhesive peptide-based hydrogels with nanoemulsions [138], encapsulating 100 nm mucosal-penetrating nanoemulsions within a self-assembling peptide hydrogel scaffold known as PuraStat. Administered orally, this nanocomposite acts as a reservoir in the stomach, enabling the controlled release of nanoemulsions into the intestine, thereby effectively alleviating intestinal inflammation.

### 4.2. Injectable Hydrogel-Based Drug Delivery

Injectable hydrogels represent a cutting-edge drug delivery system that facilitates administration through minimally invasive techniques. These hydrogels offer precise control over the kinetics and localization of drug release, making them exceptionally suited for targeted treatments across various medical conditions [139]. Injectable hydrogels require rheological properties tailored for easy administration and effective performance. They must demonstrate shear thinning behavior, reducing in viscosity under the shear stress of injection but quickly recovering once injected, facilitating both the ease of passage through needles and immediate stability within the target site [140]. Additionally, an optimal balance of viscosity and elasticity is essential—viscosity allows the hydrogel to flow smoothly during injection, while elasticity ensures it retains its structure once in place. The formulation must also account for gelation time and temperature sensitivity, ensuring the hydrogel transitions from liquid to solid at body temperature swiftly after injection. This rapid gelation at 37 °C is critical for providing enough time for injection and securing the hydrogel’s position within the target area immediately afterward. Recent advancements in hydrogel technology have demonstrated their capacity for high drug encapsulation efficiency and the simultaneous delivery of multiple therapeutic agents, leading to the effective management of cancer and other diseases [141,142].

**Figure 4:**
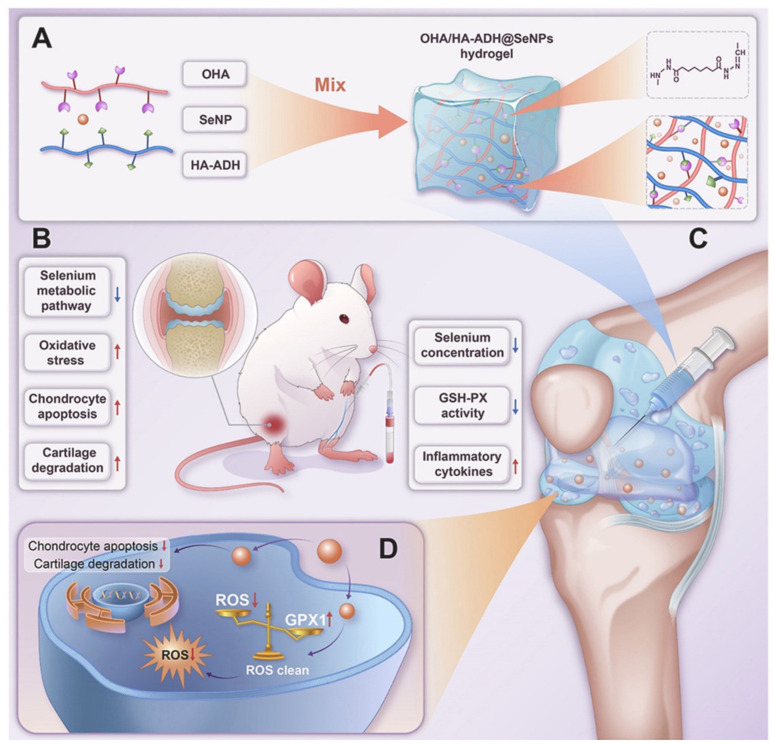
Scheme of the hydrogel-based delivery system described by Hu et al. [146]. (**A**) Synthesis of OHA/HA-ADH-gels, highlighting their chemical versatility and ability to encapsulate SeNPs. (**B**) Osteoarthritis is characterized by selenium deficiency, oxidative stress, cartilage damage, and increased inflammatory cytokines. (**C**) The gels serve as an intra-articular platform for sustained SeNPs delivery to the inflamed joint. (**D**) They mitigate cartilage degeneration by restoring redox balance and inhibiting apoptosis via GPX1 activation. Copyright *©* 2023, Elsevier Ltd.

One notable study by Kang et al. detailed the development of an injectable thermoresponsive hydrogel nanocomposite for the post-surgical management of glioblastoma multiforme (GBM) [143]. This nanocomposite incorporates drug-laden micelles and ferrimagnetic iron oxide nanocubes (wFIONs), which, upon injection into the site of tumor resection, solidify into a gel at body temperature, creating a deep intracortical depot for drug delivery. The micelles are engineered to release the drug directly to residual GBM cells, minimizing premature dispersion, while the wFIONs, under an alternating magnetic field, enhance drug permeation. Tested in an orthotopic mouse model of GBM, this hydrogel nanocomposite significantly impeded tumor progression and extended survival, showcasing its potential for effective GBM postoperative care. Lin et al. introduced a chitosan micellar self-healing hydrogel (CM hydrogel) tailored for brain tissue regeneration following intracerebral hemorrhage (ICH) stroke [144]. Formulated from phenolic chitosan (PC) and a micellar cross-linker (DPF), this hydrogel matches the mechanical properties of brain tissue and administers two model drugs with asynchronous release patterns to ICH rats, fostering behavioral recovery and equilibrium in brain movements. The CM hydrogel emerges as a novel therapeutic avenue for ICH stroke, promoting neurogenesis and angiogenesis. Wang et al. disclosed an injectable, self-reinforcing blueantagomir-21-loaded nanogel-encapsulated hydrogel (NG@antagomir-21) designed for gene delivery to repair degenerated nucleus pulposus [145], blue, which can provide sufficient mechanical support and maintain the stability of the spinal segment. Hu et al. developed an injectable hydrogel for delivering selenium nanoparticles (SeNPs) aimed at treating osteoarthritis [146]. As illustrated in Figure 4, by incorporating SeNPs into a hydrogel composed of oxidized hyaluronic acid (OHA) and hyaluronic acid-adipic acid dihydrazide (HA-ADH), they crafted a platform with minimal toxicity, self-healing capabilities, and sustained drug release properties. The hydrogel facilitates cartilage repair by scavenging reactive oxygen species (ROS) and reducing apoptosis, mainly through targeting glutathione peroxidase-1 (GPX1), a key enzyme in redox homeostasis. Their findings, demonstrated in an osteoarthritis rat model, highlight the therapeutic potential of this approach for osteoarthritis treatment, emphasizing its innovative mechanism for addressing selenium imbalances in biomaterial development for osteoarthritis therapy. Tian et al. proposed an injectable hydrogel nanostructure that enables near-infrared-controlled drug release for the photothermal and endocrine synergistic management of endometriosis [147]. Gregorio et al. developed soft, injectable, and biocompatible hydrogels, Ac-K1 and Ac-K2, incorporated with iopamidol—an iodinated contrast agent authorized for X-ray computed tomography. These hydrogels exhibit efficiency for CEST-MRI, highlighting their potential as smart MRI-detectable hydrogels [148]. Furthermore, Li et al. designed a pH-responsive injectable hydrogel composed of the octapeptide FOE, which disintegrates within the tumor microenvironment. This disintegration enhances the cellular uptake of doxorubicin through morphological transformations, thereby potentially advancing the clinical application of anti-cancer drugs [149].

Moreover, injectable hydrogels have facilitated the delivery of immunomodulatory agents, extending the duration of drug presence at the target site and thereby enhancing the immune response [150]. Wang et al. crafted a novel hydrogel vaccine, amalgamating nucleic acids (NA) to address challenges such as insufficient antigen encapsulation, inadequate immune activation, and the immunosuppressive tumor microenvironment in cancer treatment [151]. This innovative vaccine merges the chemotherapeutic agent 7-ethyl-10-hydroxycamptothecin (SN38), the immune stimulant CpG fragment, and programmed cell death ligand-1 (PD-L1) siRNA, serving as an immune checkpoint inhibitor. The hydrogel prompts immunogenic cell death, enhances antigen presentation, and fosters the maturation of dendritic cells and the infiltration of effector T lymphocytes while alleviating the immunosuppressive tumor milieu. Dai et al. introduced an ultrasound-mediated hydrogel delivery platform, HFTiDP, encapsulating a sonosensitizer (Ti-MOF-Au), a chemotherapy prodrug (PEG-TK-DOX), and the extracellular matrix solubilizing drug pirfenidone (PFD), achieving high efficacy and biosafety in localized cancer therapy by overcoming dense ECM and immunosuppressive environments in malignant solid tumors [152]. Collectively, these studies underscore the broad applicability and substantial promise of injectable hydrogels in medical treatment and drug delivery.

### 4.3. Topical Hydrogel-Based Drug Delivery

Topical delivery systems enable the active drug to make direct contact with target organs such as the skin and eyes, establishing it as a preferred route for the local administration of active compounds due to its cost-effectiveness and convenience. This method is particularly advantageous in treating conditions like wound healing and skin cancer [153]. In recent decades, a variety of topical drug delivery systems, including creams, aerosols, lotions, and powders, have been developed [154]. Hydrogel dressings, in particular, have shown promise in delivering therapeutic agents, such as biosignaling molecules and antibacterial agents, for wound care and the management of chronic wounds [155].

**Figure 5:**
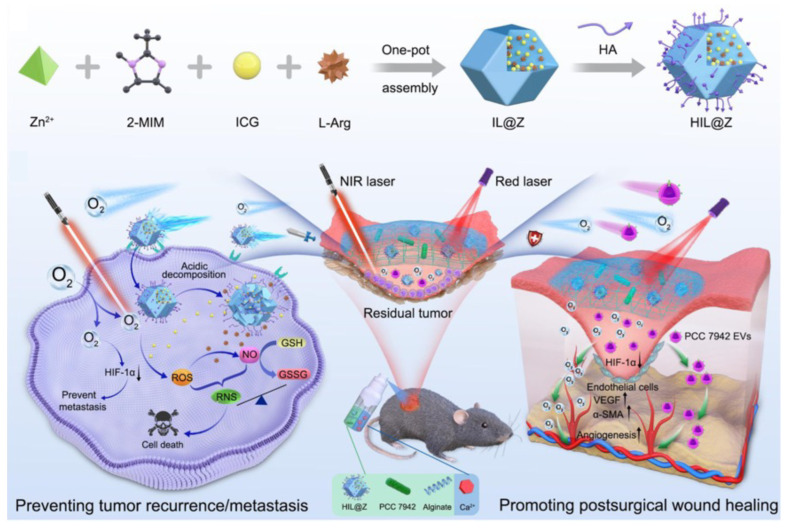
Scheme of the hydrogel-based delivery system described by Chen et al. [159]. Copyright *©* 2024, Springer Nature.

Chronic wounds frequently harbor biofilm-forming bacteria and exhibit elevated levels of oxidative stress. Existing dressings aimed at facilitating the healing of chronic wounds often necessitate supplementary interventions like photothermal irradiation or result in the accumulation of substantial, unwanted residues. Pranantyo et al. engineered a hydrogel dressing for topical application with dual functionality, featuring intrinsic antibiofilm and antioxidative properties, through a crosslinked network with integrated antibacterial cationic polyimidazolium and antioxidative N-acetylcysteine [156]. This dressing facilitated wound closure in murine diabetic wounds infected with methicillin-resistant Staphylococcus aureus or carbapenem-resistant Pseudomonas aeruginosa biofilms. Additionally, in a human skin equivalent model, the dressing promoted keratinocyte differentiation and re-epithelialization, offering a versatile and contaminant-free solution for the treatment of chronic wounds. Tan et al. reported on hydrogel dressings laden with dandelion-derived vesicles, capable of neutralizing Staphylococcus aureus exotoxins for invasive wound care [157]. Surgical site infection (SSI) is a feared complication, and developing wound dressings that effectively combat bacterial infection and promote tissue regeneration is clinically significant. Wang et al. introduced a near-infrared (NIR) light-responsive multifunctional system (PDA/Mup@DA-HA) consisting of mupirocin-loaded polydopamine nanoparticles (PDA) and dopamine-modified hyaluronic acid (DA-HA) hydrogel dressing [158]; it induces the destruction of bacterial integrity and enhances the effective release of the antibacterial drug mupirocin under near-infrared light irradiation, thereby synergistically leading to bacterial inactivation and accelerate bacteria-infected wound healing. Surgery is the main treatment modality for malignant melanoma, but the worsened hypoxic microenvironment after surgery is the source of tumor recurrence/metastasis and delayed wound healing. Chen et al. developed a sprayable hydrogel [159], that encapsulates tumor-targeted nanodrugs and photosynthetic cyanobacteria (PCC 7942) for the dual purpose of preventing tumor recurrence/metastasis and promoting wound healing after surgery as shown in Figure 5. The hydrogel works by disrupting cellular redox homeostasis in tumor cells via photodynamic therapy-induced reactions, while the photosynthetically generated oxygen from PCC 7942 not only potentiate the oxidative stress-triggered cell death to prevent local recurrence of residual tumor cells, but also block the signaling pathway of hypoxia-inducible factor 1α to inhibit their distant metastasis. Additionally, the oxygen supply and extracellular vesicles from PCC 7942 promote angiogenesis and accelerating wound healing, showing significant potential for post-surgical cancer therapy. Bao et al. crafted a multifunctional biomimetic hydrogel dressing that offers anti-infection treatment and enhances immunotherapy by reprogramming the infection-related wound microenvironment [160]. Additionally, there is some research focus on natural composites in wound healing for topical drug therapy. Zmejkoski et al. used gamma rays to synthesize nanoscale chitosan dots (ChiD) and integrate them into a bacterial cellulose (BC) polymer matrix to form a novel photosensitive protective hydrogel by using methicillin-resistant Staphylococcus aureus, demonstrating the potential against biofilm-associated infections of hydrogel, making it highly beneficial for wound healing purposes [161]. Zmejkoski et al. also developed a novel composite hydrogel for potential use as a chronic wound dressing, comprising bacterial cellulose (BC) and chitosan polymer (Chi-BC-Chi), along with chitosan nanoparticles (nChiD-BC-nChiD) [162]. Their research demonstrated excellent dressing properties, including higher porosity, increased wound fluid absorption, and accelerated cell migration, highlighting the hydrogel’s potential as an effective agent for chronic wound healing.

### 4.4. Ocular Hydrogel-Based Drug Delivery

When delivering drugs to the eye, numerous physiological challenges, including low corneal permeability, rapid tear drainage, and frequent blinking, can impede effective delivery. Consequently, conventional eye drops are swiftly expelled from the eye, leading to restricted drug absorption and diminished ocular bioavailability [163].

Additionally, hydrogels show potential as topical medications for eye diseases, with extensive ongoing research in this area. Ou et al. introduced a novel approach for treating dry eye disease using aldehyde-functionalized F127 hydrogel eye drops delivering antioxidant Cu2-xSe nanoparticles [164]. These nanoparticles, acting as superoxide dismutase and glutathione peroxidase mimics, scavenge reactive oxygen species, mitigating oxidative damage. In a dry eye mouse model, the Cu2-xSe nanoparticles showed therapeutic promise by modulating the NRF2 and p38 MAPK pathways, reducing apoptosis and inflammation, and the AF127 hydrogel eye drops demonstrating effective ocular surface adherence. This suggests a highly efficacious therapeutic strategy for dry eye disease and reactive oxygen species-related disorders. Shi et al. developed a nanozyme-thixotropic anionic hydrogel with multi-enzyme-mimicking activity for fungal keratitis treatment [165], further illustrating the promising future of topical delivery. Shi et al. developed a multi-enzyme-mimicking nanozyme-thixotropic anionic hydrogel coating (NHC) by reacting a self-synthesized polyaldehyde oligomer (PAO) with amino-functionalized hyaluronic acid (AHA) via the Schiff base reaction [165]. This hydrogel, embedding voriconazole and copper-proanthocyanidins (CuPC) nanozyme, targets the treatment of fungal keratitis. Meanwhile, Liu et al. demonstrated the use of cationic peptides [166], like Nap-FFKK, as molecular hydrogelators that spontaneously form supramolecular hydrogels within a pH range of 5–7. These hydrogels, notable for their high ocular tolerance, biocompatibility, and non-toxicity, improve corneal surface retention and adhesion through ionic interactions with ocular surface mucins, making them promising for ocular drug delivery.

In summary, the application of hydrogels in drug delivery systems has been extensively explored across oral, injectable, and topical modalities, demonstrating their unique advantages and potential for improving therapeutic outcomes. Table 2 summarizes the representative hydrogel-based drug delivery systems across various applications, detailing the delivery route, hydrogel formulation, active agent, and specific applications. These developments not only highlight the versatility of hydrogels as drug delivery vehicles but also point towards their significant role in advancing patient care and treatment efficacy across a variety of medical conditions.

## 5. Challenges and Perspectives

In the domain of drug delivery, the evolution of hydrogels from traditional chemical-based compositions to advanced supramolecular structures represents a paradigm shift. This transition has been facilitated by significant advancements in material chemistry and polymer science and complemented by cutting-edge fabrication techniques such as three-dimensional (3D) printing and microfluidics. These advanced hydrogels are engineered to possess diverse functional properties, including the ability to respond to specific stimuli, be injected directly into target sites, and offer controlled drug release kinetics tailored to individual patient needs. The capacity to engineer complex microscale and nanoscale architectures not only augments the versatility of hydrogels but also substantially amplifies their applicability in surmounting intricate delivery challenges. Despite these technological advances, the translation of hydrogel-based products from research laboratories to clinical settings has been notably slow, with 16 commercial products available for oral drug delivery, 17 for vaginal drug delivery, 16 for buccal drug delivery, and 7 for transdermal drug delivery [167]. This discrepancy underscores a prevalent gap between laboratory innovations and their practical implementation.

One of the primary obstacles impeding the clinical integration of hydrogels pertains to the regulatory and manufacturing complexities inherent to their sophisticated nature. Challenges related to storage, degradation, sterilization, and the nuanced equilibrium between material complexity and regulatory compliance have stymied their transition from laboratory settings to clinical practice. Nevertheless, the integration of hydrogels with precision medicine and the emerging field of biofabrication—particularly in developing bioinks for 3D bioprinting—heralds novel avenues of opportunity. Such advancements, aimed at forging personalized tissue constructs and refining drug delivery systems, emphasize the imperative for design simplification to enable regulatory endorsement and commercial viability while maintaining functional integrity.

As we stand at the threshold of revolutionary breakthroughs in hydrogel technology for drug delivery, the establishment of explicit design principles and the enhancement of theoretical models are paramount to optimizing drug release mechanisms and improving the predictability of therapeutic efficacy. Further exploration into novel material combinations and the mitigation of biocompatibility and immunological concerns are essential for broadening the clinical application of hydrogels. Through a concerted effort to address these challenges and fully exploit the capabilities of hydrogels, the horizon looks promising for advancements in therapeutic delivery and regenerative medicine. This evolution heralds a significant leap towards the realization of customized and more efficacious healthcare interventions.

## 6. Conclusions

Hydrogels have revolutionized the field of drug delivery, offering versatile and sophisticated platforms for targeted therapy and regenerative medicine. Despite their promising attributes, such as high water content, biocompatibility, and controlled release capabilities, the path toward their clinical adoption is fraught with regulatory, manufacturing, and biological challenges. To bridge the gap between laboratory research and clinical applications, future endeavors should focus on simplifying hydrogel designs to meet regulatory standards, developing improved theoretical models for predictable therapeutic outcomes, and exploring new material combinations to enhance biocompatibility. Embracing these strategies will propel the advancement of hydrogel technologies, paving the way for their integration into precision medicine and opening new avenues for customized and effective healthcare solutions.

## Figures and Tables

**Table 1:** Comparison of common methods used in hydrogel characterization.

Property	Methods	Advantages	Disadvantages
Mesh size	Rheological testing	Non-destructive, suitable for varied hydrogels, time-dependence analysis.	Specialized equipment needed, complex interpretation.
	Estimation from swelling ratio	Straightforward, informative.	Limited by the drawbacks of swelling ratio methods.
	Mechanical testing	Correlates with mechanical properties, diverse application.	Indirect estimation, sample preparation can be complex.
	Scattering techniques	Broad applicability, non-destructive, in-situ analysis possible.	Advanced equipment required, sensitive to sample preparation.
Swelling ratio	Weight change	Simple, cost-effective, broadly applicable.	Influenced by environmental factors, not for fast-swelling gels.
	Volumetric change	Direct measurement, effective for significant swelling.	Challenging for irregular shapes or small samples.
	Differential scanning calorimetry (DSC)	Quantitative, non-destructive.	Specialist interpretation needed, higher equipment cost.
Porosity	Estimation from swelling ratio	Straightforward, informative.	Limited by the drawbacks of swelling ratio methods.
	Mercury intrusion porosimetry	Accurate pore size and distribution, reproducible.	May alter structure, mercury is hazardous.
	SEM	Detailed surface imagery, pore size and distribution.	Dry samples only, surface-level.
	Gas adsorption	Non-destructive, good for surface area and microporosity.	Limited to surface, not suitable for all types.
	Capillary flow porometry	Provides thorough porosity data.	Requires careful choice of phases.
Microstructure	SEM	Detailed surface imagery, pore size and distribution.	Dry samples only, surface-level.
	TEM	High-resolution internal images.	Requires meticulous preparation, small coverage.
	NMR	Molecular insights, non-destructive.	Expensive, complex data interpretation.
	AFM	Nanoscale surface topology.	Surface-specific, complex sample prep.
	Confocal microscopy	3D imaging, non-destructive.	Limited penetration, fluorescence needed.
	X-ray CT	Non-destructive 3D internal imaging, high resolution.	High cost, requires contrast agents for some hydrogels.
Mechanical strength	Dynamic Mechanical Analysis (DMA)	Frequency-dependent properties.	Requires specific equipment and sample shapes.
	Rheological testing	Measures viscoelastic properties, non-destructive.	Complex interpretation, condition-sensitive.
	Tensile and compression testing	Direct strength and elasticity measurement.	Destructive, specific sample shapes necessary.
Degradability	Mass loss measurement	Direct, simple quantification.	May overlook subtle changes.
	Gel Permeation Chromatography (GPC)	Detailed profile, molecular weight insight.	Complex, requires solubilization.
	NMR	Degradation pathways at the molecular level.	Requires expensive equipment, expertise for data interpretation.
	Viscosity measurement	Indicates molecular weight changes.	Indirect, requires careful interpretation.

**Table 2:** Overview of representative hydrogel-based drug delivery systems and their applications.

Delivery Route	Hydrogels	Formulation	Active Reagent	Application
Oral	Sodium alginate hydrogel	Attach medium-chain lipids (C10) onto the surface of zeolitic imidazole framework-90 (ZIF-90), then encapsulated the nanoparticles with sodium alginate [130].	Protein.	Protein therapeutics.
	Calcium alginate hydrogel	Coating hyaluronic acid-modified selenium nanoparticles with a protective shell of calcium alginate (SA) hydrogel [131].	Selenoprotein.	Inflammatory bowel disease.
	Hyaluronic acid hydrogel	Insulin-loaded glucose-responsive nanocarriers were encapsulated into a three-dimensional hyaluronic acid hydroge [134].	Insulin.	Diabetes.
	Sodium alginate hydrogel	Zein/sodium alginate-based core-shell microspheres (Zein/SA/BG) are developed for oral delivery of Bioactive glass [135].	Bioactive glass.	Inflammatory bowel disease.
	PEG hydrogel	Synthesized via nanoprecipitation using the pH-responsive copolymers based on poly(methacrylic acid-co-methyl methacrylate)-block-poly(ethylene glycol) [136].	Antibody.	Antibody therapies.
	Sodium alginate biopolymer and poly vinyl alcohol (PVA) hydrogel	Pristine and curcumin loaded calcium alginate/poly vinyl alcohol beads (CA/PVA and CA/PVA/Cur) were prepared by an ionotropic gelation method of SA followed by crosslinking of PVA [137].	Curcumin.	Colon cancer.
	Peptide-based hydrogel	Mucopenetrating nanoemulsions of 100 nm are embedded in a scaffold constituted of the self-assembling peptide hydrogel product [138].	Cytokine.	Inflammatory bowel diseases.
Inject	Hyaluronic acid hydrogel	Oxidized hyaluronic acid (OHA) cross-linked with hyaluronic acid-adipic acid dihydrazide (HA-ADH), further incorporated with SeNPs [146].	Selenium.	Osteoarthritis.
	PLA-PEG-PLA hydrogel	Drug-loaded micelles mixed with water-dispersed ferrimagnetic iron oxide nanocubes (wFIONs) [143].	Doxorubicin.	Glioblastoma multiforme.
	Chitosan hydrogel	Chitosan micellar self-healing hydrogel (CM hydrogel) with comparable modulus to brain [144].	Curcumin.	Stroke.
	Peptide-based hydrogel	Cross-linking oxidized dextran (Ox-Dex) with MMP-2-cleavable peptide [145].	Antagomir-21.	Intervertebral disc degeneration.
	Agarose (AG) hydrogels	Polydopamine (PDA), letrozole (LTZ), and agarose (AG) hydrogels were combined to construct an near-infrared controlled drug delivery [147].	Letrozole.	Endometriosis.
	Peptide-based hydrogel	Iopamidol was loaded into Ac-K1/Ac-K2 HGs [148].	Iopamidol.	Imaging agents.
	Peptide-based hydrogel	pH-responsive ionic-complementary octapeptide FOE to delivery DOX [149].	Doxorubicin.	Cancer.
	Nucleic acid hydrogel	Crosslinking PD-L1 siRNA with a SN38- and CpG-containing Y-motif [151].	PD-L1 siRNA.	Cancer.
	Hyaluronic acid hydrogel	HA-F127@Ti-MOF-Au/PEG-TK-DOX/PFD (abbr. HFTiDP) encapsulates sonosensitizer (Ti-MOF-Au), chemotherapeutic prodrug (PEG-TK-DOX), and ECM-solubilizing drug pirfenidone (PFD) [152].	Doxorubicin.	Triple-negative breast cancer.
Topical	PEG hydrogel	Crosslinked polyethylene glycol (PEG) hydrogel tethered with highly potent antibacterial cationic polymer, polyimidazolium (PIM), and the antioxidant N-acetylcysteine (NAC) [156].	N-acetylcysteine.	Wound healing.
	Gelatin methacryloyl hydrogel	Gelatin methacryloyl hydrogel loaded dandelion-derived extracellular vesicle-like nanoparticles [157].	Dandelion-derived extracellular vesicle-like nanoparticles.	Wound healing.
	Hyaluronic acid hydrogel	Near-infrared (NIR) light-responsive multifunctional hydrogel system (PDA/Mup@DA-HA) consisting of mupirocin-loaded polydopamine nanoparticles (PDA) and dopamine-modified hyaluronic acid (DA-HA) hydrogel [158].	Mupirocin.	Bacterial infection and tissue regeneration.
	Calcium alginate hydrogel	Sprayable calcium alginate hydrogel encapsulating HIL@Z nanodrug and photosynthetic cyanobacteria [159].	Photosynthetic cyanobacteria.	Tumor recurrence/metastasis and wound healing.
	Porcine acellular dermal matrix	Hydrogel matrix is derived from porcine acellular dermal matrix and is loaded with bioactive glass nanoparticles doped with magnesium and loaded with Curcumin [160].	Curcumin.	Antimicrobial and wound healing.
	Chitosan hydrogel	Nanochitosan dots (ChiDs) were synthesized using gamma rays and encapsulated in bacterial cellulose (BC) polymer matrix [161].	Nanochitosan dots.	Chronic infections.
	Chitosan hydrogel	Hydrogels composed of bacterial cellulose (BC) with chitosan polymer (Chi)-BC-Chi and chitosan nanoparticles (nChiD) [162].	Bacterial cellulose with chitosan polymer.	Chronic infections.
Ocular	Aldehyde-functionalized F127 (AF127) hydrogel	Combines copper-selenium nanoparticles (Cu2-xSe NPs) AF127 [164].	Cu2-xse nanoparticles.	Dry eye disease.
	Hyaluronic acid hydrogel	Nanozyme-thixotropic hydrogel coating (NHC) incorporated voriconazole and copper-proanthocyanidins, self-synthesized polyaldehyde oligomer with amino functionalized hyaluronic acid [165].	Voriconazole and copper-proanthocyanidins.	Fungal keratitis.
	Peptide-based hydrogel	Nap-FFKK generate supramolecular hydrogels spontaneously in a pH value of 5-7 [166].	Nap-FFKK.	Ocular disorders.

## Data Availability

No new data were created.
